# Suicide risk among individuals diagnosed with cancer during versus before the COVID-19 pandemic: a nationwide population-based study

**DOI:** 10.1093/jjco/hyaf110

**Published:** 2025-07-08

**Authors:** Ken Kurisu, Maiko Fujimori, Saki Harashima, Tatsuo Akechi, Kazuhiro Yoshiuchi, Yosuke Uchitomi

**Affiliations:** Department of Cancer Survivorship and Digital Medicine, Jikei University School of Medicine, Tokyo, Japan; Division of Survivorship Research, National Cancer Center Institute for Cancer Control, Tokyo, Japan; Department of Stress Sciences and Psychosomatic Medicine, Graduate School of Medicine, The University of Tokyo, Tokyo, Japan; Division of Survivorship Research, National Cancer Center Institute for Cancer Control, Tokyo, Japan; Division of Survivorship Research, National Cancer Center Institute for Cancer Control, Tokyo, Japan; Department of Stress Sciences and Psychosomatic Medicine, Graduate School of Medicine, The University of Tokyo, Tokyo, Japan; Department of Psychiatry and Cognitive-Behavioral Medicine, Nagoya City University, Nagoya, Japan; Department of Stress Sciences and Psychosomatic Medicine, Graduate School of Medicine, The University of Tokyo, Tokyo, Japan; Department of Cancer Survivorship and Digital Medicine, Jikei University School of Medicine, Tokyo, Japan

**Keywords:** cancer, COVID-19 pandemic, suicide, loneliness, social isolation

## Abstract

This study aimed to assess suicide risk among individuals diagnosed with cancer shortly after the onset of the COVID-19 pandemic compared to those diagnosed earlier. Data were obtained from the National Cancer Registry in Japan, which included 4 711 540 individuals diagnosed between January 2016 and June 2020. Standardized mortality ratios (SMRs) for suicide within 6 months of diagnosis were quantified using the general population as a reference. Those diagnosed between April and June 2020 had an SMR of 3.93 (95% confidence interval: 3.10–4.91), higher than those diagnosed in earlier periods. Additionally, multivariate Poisson regression showed an adjusted relative risk of 1.30 (95% confidence interval: 1.03–1.63) for individuals diagnosed during the pandemic compared to earlier periods. These findings suggest that individuals diagnosed with cancer shortly after the onset of the COVID-19 pandemic had a higher suicide risk than those diagnosed earlier.

## Teaser

This analysis using the National Cancer Registry suggests that individuals diagnosed with cancer shortly after the onset of the COVID-19 pandemic had a higher suicide risk than those diagnosed earlier.

## Introduction

People diagnosed with cancer show high rates of depression and suicide worldwide [1, 2]. In Japan, nationwide data from the National Cancer Registry also indicate elevated suicide risk in this population, along with regional disparities [3–5]. Efforts have been made to develop suicide prevention strategies targeting individuals with cancer [6, 7].

The COVID-19 pandemic has increased psychological distress [8], loneliness, and social isolation [9]. It has also caused changes in suicide rates [10, 11] and a rise in psychiatric conditions such as eating disorders in Japan [12].

Studies have reported that individuals with cancer experienced greater loneliness, psychological distress, depression, and anxiety during the pandemic [13–15]. Cross-sectional research has examined suicidal ideation and attempts during that period [16]. While these findings suggest worsened mental health among individuals with cancer during the pandemic, no studies have examined changes in suicide rates before and during the pandemic.

Recent data from Japan’s National Cancer Registry, which includes all individuals diagnosed with cancer nationwide, allow for the analysis of people diagnosed in early 2020. This study aimed to assess suicide risk among people diagnosed with cancer shortly after the onset of the COVID-19 pandemic—a period characterized by heightened stress—and to compare their risk with those diagnosed before the pandemic.

## Materials and methods

### Ethical approval

The study was approved by the Institutional Review Board of the National Cancer Center Japan (approval number: 2018-233). The requirement for informed consent was waived owing to the use of anonymous data.

### Study participants

This study used data from the National Cancer Registry, which covers all individuals diagnosed with cancer in Japan since January 2016 [3–5, 17]. The current dataset includes records of death through 31 December 2020. Therefore, those diagnosed between January 2016 and June 2020 were included, and the maximum observation period was set to 6 months.

In April 2020, the Japanese government declared a state of emergency due to an exponential increase in the number of COVID-19 infections, and this time point has been used as a cutoff in previous literature [12]. Therefore, those diagnosed between April and June 2020 were classified as the pandemic-diagnosis group, whereas those diagnosed before April 2020 were classified as the pre-pandemic-diagnosis group.

Following previous studies [3–5, 17], we excluded individuals diagnosed by autopsy or death certificate, those residing outside Japan, and those with missing data on age, sex, address, or date of diagnosis.

The outcome was death by suicide, defined using the International Classification of Diseases, 10th Edition codes X60-X84 and Y87.0.

### Statistical analyses

Standardized mortality ratios (SMRs) were calculated for each diagnostic period, following our previous studies and protocol [3–5, 17]. The observed number of suicides within 6 months after diagnosis was calculated using data from the National Cancer Registry. For each individual, the expected suicide rate during the corresponding survival period was estimated using data from the general population, matched by age, sex, and prefecture of residence, based on Vital Statistics and Population Estimates. Summing these rates yielded the expected number of suicides in the study population. Patients registered with zero months of survival in the dataset were assigned a value of 0.5 months. The SMR was defined as the ratio of observed to expected suicides. Confidence intervals (CIs) were calculated using Byar’s method.

A multivariable Poisson regression model was developed to examine differences in SMRs between the pandemic- and pre-pandemic-diagnosis groups. Following previous research [3, 4], the individual-level dataset with binary mortality outcomes was aggregated into a cluster-level dataset with count outcomes. The log-transformed expected suicide counts for each cluster were included as an offset in the model. Accordingly, the relative risk from the model represented the ratio of SMRs between groups. Age, sex, presence of multiple primary cancers, primary tumor site, and cancer extension were included as covariates, consistent with previous studies [3, 4].

Additional Poisson regression models were developed for sensitivity analyses: (i) a model comparing individuals diagnosed between January and June 2020 with those diagnosed before 2020, (ii) a model including January–March 2020 as a separate category, and (iii) a model containing only individuals diagnosed between April and June of each year, comparing 2020 with previous years.

All analyses were conducted using R (version 4.4.2) with the ‘epiR’ package (version 2.0.77). Statistical significance was set at *P* < 0.05.

## Results

**Figure 1:**
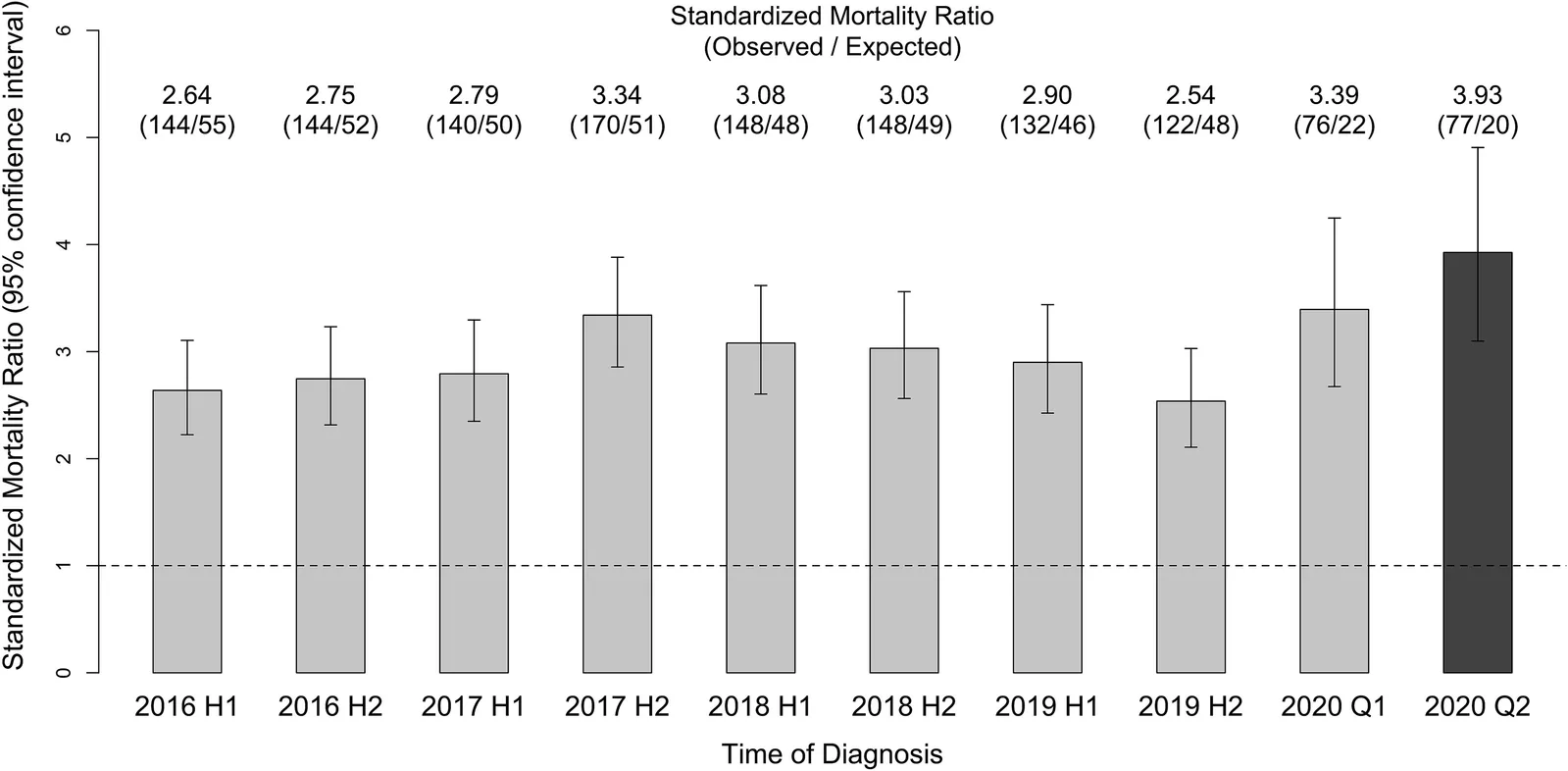
Standardized mortality ratio for suicide within 6 months after cancer diagnosis, by time of diagnosis. H1 and H2 represent January–June and July–December, respectively; Q1 and Q2 represent January–March and April–June, respectively.

The analysis included 4 711 540 individuals (see Supplementary Table 1 for descriptive statistics). Figure 1 summarizes the 6-month SMRs for each diagnosis period. Among those diagnosed between April and June 2020, the SMR was 3.93 (95% CI: 3.10–4.91), indicating a modest increase compared with earlier periods. Sex-specific SMRs during this period were 4.01 (95% CI: 3.04–5.19) for male individuals and 3.71 (95% CI: 2.26–5.73) for female individuals, with no substantial sex difference observed.

Table 1 summarizes the relative risk estimated from the Poisson regression model. In Model 1, the pandemic-diagnosis group had a relative risk of 1.30 (95% CI: 1.03–1.63), with the pre-pandemic-diagnosis group as a reference. Coefficients for other covariates are shown in Supplementary Table 2 and were similar to those reported in the previous study [3]. Sensitivity analyses using alternative groupings (Models 2 and 3) and a model including only individuals diagnosed between April and June of each year (Model 4) also showed significantly higher relative risks for the pandemic period.

**Table 1:** Relative risk by time of cancer diagnosis from multivariate Poisson regression models.

Time of diagnosis	Total number of suicide (N)	Relative risk (95% confidence interval)	*P*-value
Model 1 (primary)			
January 2016–March 2020	1224	1.00 (reference)	
April–June 2020	77	1.30 (1.03 to 1.63)	**0.028**
Model 2			
January 2016–December 2019	1148	1.00 (reference)	
January–June 2020	153	1.24 (1.05 to 1.47)	**0.013**
Model 3			
January 2016–December 2019	1148	1.00 (reference)	
January–March 2020	76	1.18 (0.93 to 1.49)	0.17
April–June 2020	77	1.31 (1.04 to 1.65)	**0.023**
Model 4			
April–June in 2016–2019	274	1.00 (reference)	
April–June 2020	77	1.40 (1.09 to 1.80)	**0.009**

All models were adjusted for age, sex, presence of multiple primary cancers, primary tumor site, and cancer extension. Bold entries indicate statistically significant at *P* < 0.05.

## Discussion

Using data from the National Cancer Registry, we assessed suicide risk among individuals diagnosed with cancer during the COVID-19 pandemic. The SMR was higher in individuals diagnosed during the pandemic than in those diagnosed earlier. This elevated risk persisted after adjusting for covariates and remained consistent across different categorizations of diagnostic periods. To the best of our knowledge, this is the first report to compare suicide risk between individuals diagnosed with cancer during the COVID-19 pandemic and those diagnosed earlier.

These findings indicate that suicide risk in individuals with cancer, which is consistently higher than in the general population, increased further during the pandemic and exceeded the fluctuations observed in the general population [10, 11]. Previous studies have reported increased psychological distress and loneliness among individuals with cancer during the pandemic [13–15]. SARS-CoV-2 infection itself may also have partially influenced mental health [18]. In addition, the psychological states of individuals with life-threatening illnesses and those living through the pandemic have been discussed within the framework of terror management theory [19, 20]. These perspectives may help inform future research aimed at understanding the mechanisms behind our findings.

In future pandemics or disasters, greater attention to the mental health of individuals newly diagnosed with cancer will be essential. As discussed above, potential mechanisms underlying the increase in suicide risk may include heightened loneliness and social isolation among people with cancer during the COVID-19 pandemic [13, 14]. The present findings imply that such loneliness or social isolation may have been more pronounced than in the general population, leading to an even greater increase in suicide risk among people with cancer. Various interventions for loneliness have been explored [21–23], and these approaches may inform strategies to support vulnerable populations during future crises.

This study has several limitations. First, the observation period was limited to 6 months following diagnosis and thus does not capture outcomes after societal adaptation to the pandemic, such as vaccine rollout and other mitigation efforts. Second, we were unable to assess the specific factors contributing to the increased suicide risk or determine why the magnitude of this increase exceeded that observed in the general population. Finally, the dataset did not include information on preexisting mental disorders, household composition, or socioeconomic status. These factors should be considered in future research.

In conclusion, individuals diagnosed with cancer shortly after the onset of the COVID-19 pandemic had an ~1.3-fold higher suicide SMR than those diagnosed before the pandemic. These findings underscore the importance of addressing mental health in individuals newly diagnosed with cancer during future pandemics or disasters. Further research with extended follow-up is warranted to examine longer-term trends.

## Supplementary Material

20250517_Supplementary_hyaf110

## Data Availability

The dataset analyzed in this study, obtained in 2024, was used with permission from the National Cancer Registry Information Desk of the National Cancer Center. This dataset cannot be shared; however, anyone can access it by sending an application along with their study protocol to the National Cancer Registry Information Desk.
