# Correction to: Suicide risk among individuals diagnosed with cancer during versus before the COVID-19 pandemic: a nationwide population-based study

**DOI:** 10.1093/jjco/hyag148

**Published:** 2026-09-05

This is a correction to: Ken Kurisu, Maiko Fujimori, Saki Harashima, Tatsuo Akechi, Kazuhiro Yoshiuchi, Yosuke Uchitomi, Suicide risk among individuals diagnosed with cancer during versus before the COVID-19 pandemic: a nationwide population-based study, *Japanese Journal of Clinical Oncology*, Volume 55, Issue 10, October 2025, Pages 1194–1197, https://doi.org/10.1093/jjco/hyaf110

In the originally published version of this manuscript, the numbers and proportions of patients with and without multiple primary tumors were inadvertently reversed within Supplementary Table 1.

This error has been corrected within the supplementary file.

**Table:** 

Variable	Diagnosed before April 2020 (N = 4,492,352)	Diagnosed April–June 2020 (N=219,188)
Age, N (%) 0–39 40–49 50–59 60–69 70–79 ≥80	16,9147 (3.8) 29,4104 (6.5) 451,813 (10.1) 1,022,107 (22.8) 1,396,962 (31.1) 1,158,219 (25.8)	8,223 (3.8) 13,823 (6.3) 21,836 (10.0) 42,811 (19.5) 71,703 (32.7) 60,792 (27.7)
Sex, N (%) Male Female	2,455,205 (54.7) 2,037,147 (45.3)	118,447 (54.0) 100,741 (46.0)
Multiple primary tumors, N (%) Present Absent	560,523 (12.5) 3,931,829 (87.5)	19,092 (8.7) 200,096 (91.3)
Primary tumor site, N (%) Head and neck Esophagus Stomach Colon Rectum Liver and intrahepatic bile ducts Gallbladder and other biliary tract Pancreas Lung and bronchus Skin Breast Cervix uteri Corpus uteri Ovary Prostate Bladder Kidney and urinary organs Brain and other parts of the CNS Thyroid Malignant lymphoma Multiple myeloma Leukemia Other	104,486 (2.3) 103,650 (2.3) 499,837 (11.1) 525,445 (11.7) 243,899 (5.4) 150,554 (3.4) 86,856 (1.9) 164,839 (3.7) 486,409 (10.8) 123,430 (2.7) 442,462 (9.8) 143,717 (3.2) 68,880 (1.5) 52,307 (1.2) 366,792 (8.2) 169,498 (3.8) 113,793 (2.5) 23,747 (0.5) 72,072 (1.6) 139,467 (3.1) 30,466 (0.7) 56,806 (1.3) 322,940 (7.2)	5,533 (2.5) 4,669 (2.1) 20,394 (9.3) 24,726 (11.3) 11,394 (5.2) 7,106 (3.2) 4,672 (2.1) 9,634 (4.4) 243,66 (11.1) 5,614 (2.6) 21,902 (10.0) 6,926 (3.2) 3,706 (1.7) 2,880 (1.3) 16,517 (7.5) 9,351 (4.3) 5,964 (2.7) 1,239 (0.6) 3,117 (1.4) 8,140 (3.7) 1,554 (0.7) 3,193 (1.5) 16,591 (7.6)
Extensions, N (%) Localized Regional Metastatic Unknown/other	2,312,588 (51.5) 885,727 (19.7) 717,543 (16.0) 576,494 (12.8)	105,766 (48.3) 46,102 (21.0) 41,471 (18.9) 25,849 (11.8)

Abbreviations: CNS, central nervous system

