# Supplementary material for: Suicide risk among individuals diagnosed with cancer during versus before the COVID-19 pandemic: a nationwide population-based study
Source: Jpn J Clin Oncol. 2025 Jul 8;55(10):1194–7. doi: 10.1093/jjco/hyaf110 (PMC12501966; doi:10.1093/jjco/hyaf110)
Supplement: 20250517_Supplementary_hyaf110 [file 20250517_supplementary_hyaf110.docx]

**Supplementary Table 1. Descriptive statistics of individuals diagnosed with cancer before and during the COVID-19 pandemic in Japan**

| Variable | Diagnosed before April 2020  (N = 4,492,352) | Diagnosed April–June 2020  (N=219,188) |
| --- | --- | --- |
| Age, N (%)  0–39  40–49  50–59  60–69  70–79  ≥80 | 16,9147 (3.8)  29,4104 (6.5)  451,813 (10.1)  1,022,107 (22.8)  1,396,962 (31.1)  1,158,219 (25.8) | 8,223 (3.8)  13,823 (6.3)  21,836 (10.0)  42,811 (19.5)  71,703 (32.7)  60,792 (27.7) |
| Sex, N (%)  Male  Female | 2,455,205 (54.7)  2,037,147 (45.3) | 118,447 (54.0)  100,741 (46.0) |
| Multiple primary tumors, N (%)  Absent  Present | 560,523 (12.5)  3,931,829 (87.5) | 19,092 (8.7)  200,096 (91.3) |
| Primary tumor site, N (%)  Head and neck  Esophagus  Stomach  Colon  Rectum  Liver and intrahepatic bile ducts  Gallbladder and other biliary tract  Pancreas  Lung and bronchus  Skin  Breast  Cervix uteri  Corpus uteri  Ovary  Prostate  Bladder  Kidney and urinary organs  Brain and other parts of the CNS  Thyroid  Malignant lymphoma  Multiple myeloma  Leukemia  Other | 104,486 (2.3)  103,650 (2.3)  499,837 (11.1)  525,445 (11.7)  243,899 (5.4)  150,554 (3.4)  86,856 (1.9)  164,839 (3.7)  486,409 (10.8)  123,430 (2.7)  442,462 (9.8)  143,717 (3.2)  68,880 (1.5)  52,307 (1.2)  366,792 (8.2)  169,498 (3.8)  113,793 (2.5)  23,747 (0.5)  72,072 (1.6)  139,467 (3.1)  30,466 (0.7)  56,806 (1.3)  322,940 (7.2) | 5,533 (2.5)  4,669 (2.1)  20,394 (9.3)  24,726 (11.3)  11,394 (5.2)  7,106 (3.2)  4,672 (2.1)  9,634 (4.4)  243,66 (11.1)  5,614 (2.6)  21,902 (10.0)  6,926 (3.2)  3,706 (1.7)  2,880 (1.3)  16,517 (7.5)  9,351 (4.3)  5,964 (2.7)  1,239 (0.6)  3,117 (1.4)  8,140 (3.7)  1,554 (0.7)  3,193 (1.5)  16,591 (7.6) |
| Extensions, N (%)  Localized  Regional  Metastatic  Unknown/other | 2,312,588 (51.5)  885,727 (19.7)  717,543 (16.0)  576,494 (12.8) | 105,766 (48.3)  46,102 (21.0)  41,471 (18.9)  25,849 (11.8) |

Abbreviations: CNS, central nervous system

**Supplementary Table 2. Relative risks for all covariates in Model 1**

| Variable | Relative Risk  (95% confidence interval) | P-value |
| --- | --- | --- |
| Diagnosis Time |  |  |
| Before the pandemic (January 2016 – March 2020) | 1.00 (reference) |  |
| During the pandemic (April – June 2020) | 1.30 (1.03 to 1.63) | 0.028 |
| Age |  |  |
| 0–39 | 0.78 (0.48 to 1.26) | 0.31 |
| 40–49 | 0.94 (0.69 to 1.29) | 0.71 |
| 50–59 | 1.00 (reference) |  |
| 60–69 | 1.11 (0.90 to 1.37) | 0.32 |
| 70–79 | 1.29 (1.06 to 1.57) | 0.013 |
| ≥80 | 1.01 (0.82 to 1.25) | 0.89 |
| Sex |  |  |
| Male | 1.00 (reference) |  |
| Female | 1.02 (0.88 to 1.18) | 0.80 |
| Multiple primary tumors |  |  |
| Absent | 1.00 (reference) |  |
| Present | 0.59 (0.48 to 0.71) | <0.01 |
| Primary tumor site |  |  |
| Head and neck | 1.64 (1.18 to 2.27) | <0.01 |
| Esophagus | 2.05 (1.53 to 2.74) | <0.01 |
| Stomach | 1.23 (0.98 to 1.53) | 0.08 |
| Colon | 1.00 (reference) |  |
| Rectum | 1.03 (0.78 to 1.38) | 0.82 |
| Liver and intrahepatic bile ducts | 0.86 (0.59 to 1.26) | 0.44 |
| Gallbladder and other biliary tract | 1.00 (0.65 to 1.52) | 0.99 |
| Pancreas | 1.39 (1.03 to 1.87) | 0.031 |
| Lung and bronchus | 0.96 (0.76 to 1.22) | 0.74 |
| Skin | 0.97 (0.63 to 1.48) | 0.88 |
| Breast | 1.38 (1.03 to 1.84) | 0.031 |
| Cervix uteri | 1.02 (0.59 to 1.78) | 0.93 |
| Corpus uteri | 1.02 (0.53 to 1.96) | 0.95 |
| Ovary | 2.15 (1.29 to 3.57) | <0.01 |
| Prostate | 0.69 (0.53 to 0.89) | <0.01 |
| Bladder | 1.40 (1.04 to 1.88) | 0.028 |
| Kidney and urinary organs | 1.25 (0.88 to 1.77) | 0.21 |
| Brain and other parts of the CNS | 1.12 (0.49 to 2.55) | 0.79 |
| Thyroid | 0.46 (0.22 to 1.00) | 0.049 |
| Malignant lymphoma | 0.89 (0.62 to 1.27) | 0.51 |
| Multiple myeloma | 1.05 (0.58 to 1.90) | 0.87 |
| Leukemia | 0.86 (0.51 to 1.46) | 0.58 |
| Other | 0.79 (0.58 to 1.06) | 0.12 |
| Extension of tumors |  |  |
| Localized | 1.00 (reference) |  |
| Regional | 1.56 (1.35 to 1.81) | <0.01 |
| Metastatic | 2.27 (1.95 to 2.65) | <0.01 |
| Unknown/other | 2.10 (1.75 to 2.53) | <0.01 |
